# Implementation of Flip-Chip Microbump Bonding between InP and SiC Substrates for Millimeter-Wave Applications

**DOI:** 10.3390/mi13071072

**Published:** 2022-07-05

**Authors:** Jongwon Lee, Jae Yong Lee, Jonghyun Song, Gapseop Sim, Hyoungho Ko, Seong Ho Kong

**Affiliations:** 1 Nano Convergence Technology Division, National Nanofab Center, Daejeon 34141, Korea; jhsong@nnfc.re.kr (J.S.); gssim@nnfc.re.kr (G.S.); 2 School of Electronic and Electrical Engineering, Kyungpook National University, Daegu 41566, Korea; cheersssss@naver.com; 3 Department of Electronics Engineering, Chungnam National University, Daejeon 34134, Korea

**Keywords:** InP, SiC, flip-chip bonding, millimeter wave, heterogeneous integration

## Abstract

Flip-chip microbump (μ-bump) bonding technology between indium phosphide (InP) and silicon carbide (SiC) substrates for a millimeter-wave (mmW) wireless communication application is demonstrated. The proposed process of flip-chip μ-bump bonding to achieve high-yield performance utilizes a SiO_2_-based dielectric passivation process, a sputtering-based pad metallization process, an electroplating (EP) bump process enabling a flat-top μ-bump shape, a dicing process without the peeling of the dielectric layer, and a SnAg-to-Au solder bonding process. By using the bonding process, 10 mm long InP-to-SiC coplanar waveguide (CPW) lines with 10 daisy chains interconnected with a hundred μ-bumps are fabricated. All twelve InP-to-SiC CPW lines placed on two samples, one of which has an area of approximately 11 × 10 mm^2^, show uniform performance with insertion loss deviation within ±10% along with an average insertion loss of 0.25 dB/mm, while achieving return losses of more than 15 dB at a frequency of 30 GHz, which are comparable to insertion loss values of previously reported conventional CPW lines. In addition, an InP-to-SiC resonant tunneling diode device is fabricated for the first time and its DC and RF characteristics are investigated.

## 1. Introduction

Because the InP substrate is lattice-matched with InGaAs materials featuring a high electron mobility of more than 8000 cm^2^/vs, the InP-substrate-based low-noise amplifier (LNA) and power amplifier (PA) monolithic integrated circuits (MICs) using InGaAs high-electron-mobility transistor (HEMT) and heterojunction bipolar transistor (HBT) devices have operated at high operating frequencies above the millimeter-wave (mmW) regime [1,2]. Because the SiC substrate enables the growth of gallium nitride (GaN) materials exhibiting a high electric breakdown field of 3.3 × 10^6^ V/cm, SiC-substrate-based mmW PA MICs using GaN HEMTs have shown high RF power performance [3]. By placing the SiC substrate as the first layer and the InP substrate as the second layer, an InP/GaN three-dimensional (3D) integration structure can be implemented, leading to high-performance mmW MICs and transceiver frontends [4,5]. The InP/GaN 3D structure can basically achieve a higher chip density and lower interconnection resistance compared to those of two-dimensional (2D) structures [4,5,6], and can enhance the current drivability of InGaAs HEMT and HBT devices owing to the excellent thermal conductivity of the SiC substrate (4.9 W/cmk) [7,8,9,10], resulting in an improved RF power and frequency performance of the mmW MICs or transceiver frontends.

An appropriate bonding method should be selected to realize the mmW InP/GaN 3D structure. Wire bonding, direct wafer bonding, and microbump (μ-bump) bonding for a heterogeneous integration have been utilized. Although wire bonding is easily accessible to researchers, it causes severe system performance degradation due to significant signal loss in the mmW band or higher, arising from the lengthening of interconnect lines [11]. Although direct wafer bonding makes it possible to achieve a high chip density in the MIC and transceiver owing to the utilization of a sequential fabrication process after the direct bonding followed by substrate removal, the substrate removal process for leaving active thin-film layers requires a high degree of fabrication proficiency [7,8,9,10,12]. On the other hand, μ-bump bonding consisting of electro-plated copper (Cu) pillar bumps and solder bonding [13,14] is a mature process technology that has been used in commercial 3D stacking memory products [14,15], and thereby it can be introduced to implement mmW 3D MICs and transceiver systems reproducibly. With the improvement of the alignment accuracy of bonding equipment, it is possible to densely form μ-bumps with a diameter (or width) of only a few to a few tens of micrometers [14,16]. Recently, heterogeneous μ-bump bonding technologies concerning various substrates, such as InP-to-SiC [5], InP-to-Si [17], and AlN/diamond-to-Si [18], have been reported for utilization in mmW wireless communication applications. However, [5] did not disclose any measurement results for bonded samples and [17] showed the RF measurement results for only a bonded HBT device, which are presumed to be implemented by a few to a few tens of μ-bump connections. The authors of [18] also measured only an interconnect line with four μ-bumps. To apply bump bonding technology to mmW ICs and transceiver systems, the process methodology and the implementation results of the μ-bump bonding technology to implement a much larger number of μ-bumps should be presented.

In this work, we report a flip-chip μ-bump bonding technology between InP and SiC substrates for mmW wireless communication applications. A process methodology for InP-to-SiC flip-chip μ-bump bonding with high-yield characteristics while being easily accessible to researchers is proposed. By utilizing the bonding process, 10 mm long InP-to-SiC coplanar waveguide (CPW) lines interconnected with a hundred μ-bumps were fabricated. The fabricated InP-to-SiC CPW lines showed uniformly good performance with an insertion loss deviation within 10% and an average insertion loss of 0.25 dB/mm at a frequency of 30 GHz, which are comparable to insertion loss values of previously reported conventional CPW lines. In addition, an InP RTD device was flip-chip bonded with a SiC substrate for the first time and its DC and RF performance was investigated through a comparison with the corresponding measures of a conventional InP RTD device.

## 2. Structure Design and Fabrication

### 2.1. Structure Design of a Flip-Chip μ-Bump Bonding Technology between InP and SiC Substrates

**Figure 1:**
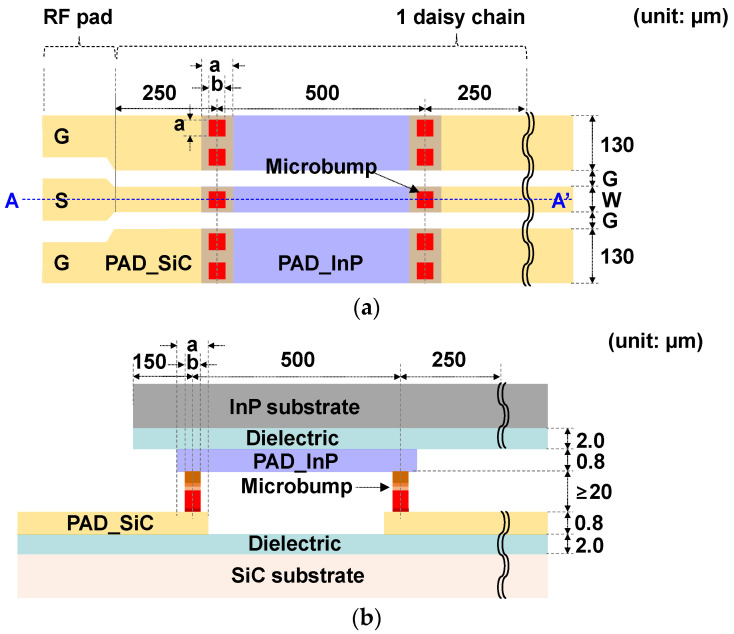
The structure of flip-chip microbump bonding technology for coplanar waveguide (CPW) interconnection between InP and SiC substrates: (**a**) a floor plan; (**b**) a cross-sectional view at point A–A’ in Figure 1a.

The designed structure of a flip-chip μ-bump bonding technology for a CPW interconnection between InP and SiC substrates is shown in Figure 1. To minimize the signal loss of the CPW line escaping from PAD metals (PAD_InP and PAD_SiC in Figure 1) to the substrates, semi-insulating InP and SiC substrates were chosen. An InP substrate, provided by JZ Nippon Mining & Metal Corporation, with a 3-inch diameter, a thickness of approximately 610 μm, and a resistivity of more than 1 × 10^7^ Ω·cm was used. A 4H-SiC substrate with a 4-inch diameter, a thickness of approximately 510 μm, and a resistivity over 1 × 10^7^ Ω·cm, provided by Synlight Crystal Co., Ltd. Hebei, China, was used. A dielectric layer of SiO_2_ or BCB (Cyclotene 3022-46 resin) with a thickness of 2 μm was inserted between the PAD metals and the substrates for device passivation and planarization, considering the ultimate integration of transistors such as HEMTs and HBTs in the future, as well as for the minimization of the signal loss from the PAD metals to substrates, as shown in Figure 1b. A Ti/Au material was used as PAD metals with the thickness set to 0.8 μm, which corresponded to the maximum limit of the sputtering equipment used in this work. The μ-bump metal consisted of Cu/Ni/SnAg and its height was set to 20 μm or more to prevent any bonding failure caused by the fragile nature of the InP substrates. From an S-parameter simulation for the flip-chip-bonded CPW line between InP and SiC substrates using the advanced design system (ADS) momentum simulator, it was determined that the signal width (W) and the gap (G) of the CPW to have a characteristic impedance of 50 Ω in the mmW frequency range of more than 30 GHz were 60 and 40 μm, respectively. The length of the μ-bump pad (‘a’ in Figure 1) was set as 60 μm, the same as W. The size of the μ-bump (‘b’ in Figure 1) was set in the range of 25 to 40 μm by comprehensively considering several phenomena such as the alignment accuracy of the flip-chip bonder equipment, the SnAg overflow in the bonding process, and the increase in the insertion loss that occurs when the bump size is quite small compared to the bump-pad length.

### 2.2. Fabrication of a Flip-Chip μ-Bump Bonding Process between InP and SiC Substrates

**Figure 2:**
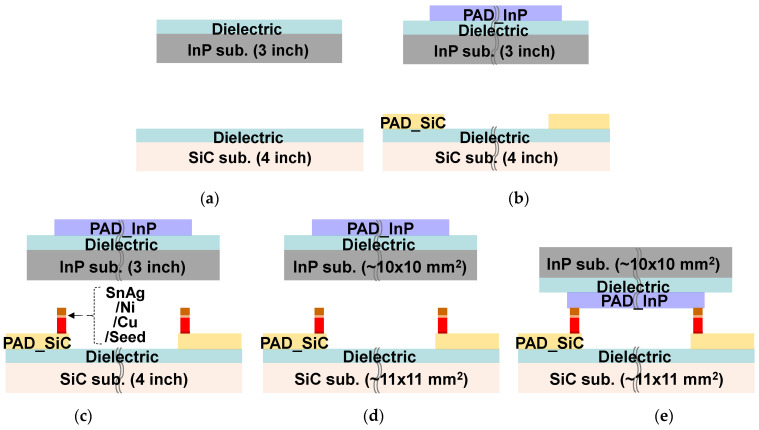
Cross-sectional view of the process flow for flip-chip microbump (μ-bump) bonding technology between InP and SiC substrates: (**a**) dielectric layer deposition; (**b**) formation of PAD metal; (**c**) formation of microbump metal; (**d**) dicing; (**e**) flip-chip SnAg-to-Au solder bonding.

Figure 2 shows a cross-sectional view of the process flow of the flip-chip μ-bump bonding for the CPW interconnection between InP and SiC substrates, which entails a dielectric layer deposition (Figure 2a), the formation of PAD metal (Figure 2b), formation of μ-bump (Figure 2c), dicing (Figure 2d), and flip-chip SnAg-to-Au solder bonding (Figure 2e).

**Figure 3:**
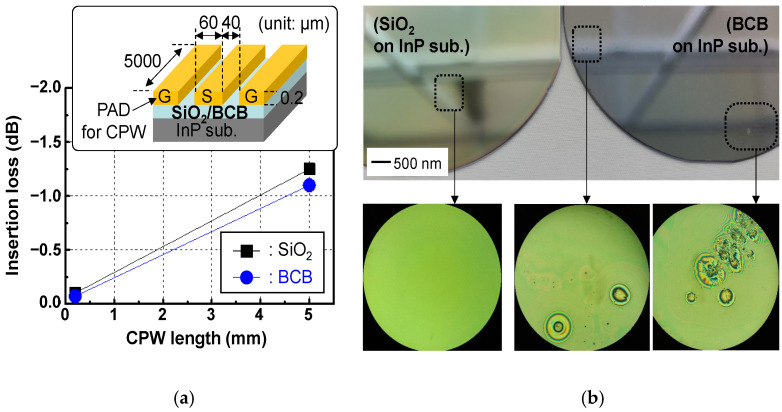
Implementation results of coplanar waveguides (CPWs) with different dielectric layers of SiO_2_ and BCB: (**a**) measured insertion loss of fabricated SiO_2_- and BCB-based coplanar waveguide (CPW) lines. The inset shows a structural diagram of the fabricated CPW lines; (**b**) microscope images immediately after deposition of SiO_2_ and BCB layers on InP substrates.

The process for the deposition of the dielectric layer, seen in a conceptual diagram of Figure 2a, was conducted. To find the appropriate material for the dielectric layer, two kinds of materials with a low dielectric constant, SiO_2_ and BCB, were tested. A 2.5 μm thick BCB was deposited on the InP substrate via spin-coating with spinner system equipment and cured at 210 °C in vacuum oven equipment with a N_2_ atmosphere. Additionally, a 2 μm thick SiO_2_ layer was deposited on the InP substrate using plasma-enhanced chemical vapor deposition (PECVD) at a temperature of 300 °C. After a 0.2 μm thick Ti/Au PAD metal for the CPW line formation was deposited on both the BCB-based and SiO_2_-based InP substrates, as shown in the inset in Figure 3a, an S-parameter measurement was conducted at a frequency of 15 GHz. As a result, the insertion loss of the BCB-based and SiO_2_-based CPW lines was 0.24 and 0.29 dB/mm at 15 GHz, respectively, as shown in Figure 3a. The lower insertion loss of the BCB-based CPW was attributed to the dielectric constant of the BCB (~2.5) being lower than that of the SiO_2_ (~3.8). Even though the BCB layer was superior to the SiO_2_ layer in terms of mmW performance, a problem was found, where the surface of the BCB layer was dirty enough to adversely affect the process yield, in contrast to the clean surface condition of the SiO_2_ layer, as shown in Figure 3b. This yield issue of BCB was dependent on the size of the substrate, which occurred when the size of the substrate was increased to 3 inches or larger. It was determined that the cause of the issue was that the BCB solution sprayed through the dropper adhered to the wall of the spinner equipment during the spinning process, and then fell off and adhered to the wafer again. Consequently, to establish a high-yield process technology, the SiO_2_ layer with a thickness of 2 μm was used as the dielectric layer. We noted that the yield issue of BCB may have been limited by our facility at this time, and could be sufficiently improved through the optimization of the spinner-based BCB process.

**Figure 4:**
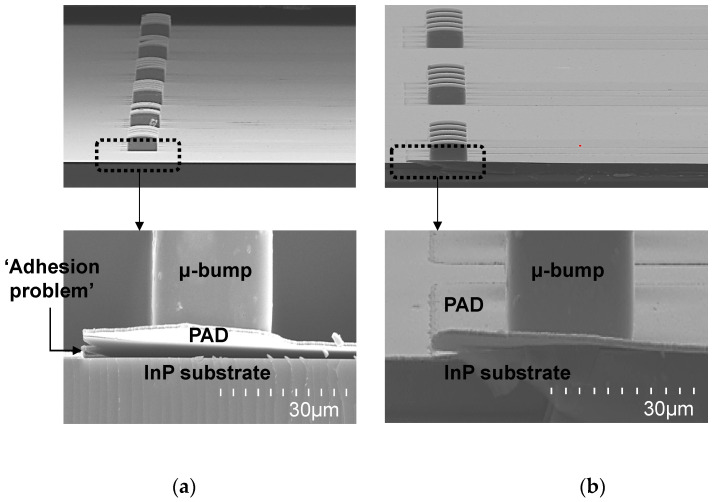
SEM images of PAD metals: (**a**) PAD metal based on an evaporation and lift-off process; (**b**) PAD metal based on a sputtering and metal etching process.

The process for the formation of the PAD metal was conducted on both SiO_2_-deposited InP and SiC substrates, as shown in Figure 2b. The process methodology of the evaporation and lift-off was first applied and the detailed process flow was as follows: A cleaning process was conducted using acetone/IPA/DI solutions. A photolithography process with a critical dimension (CD) of 60 μm and an undercut slope was performed using an NR93000PY negative photoresist (PR) and EVG640 contact aligner. The Ti/Au PAD metal with a thickness of 400/8000 Å was evaporated using the KVET-C500200 evaporator. A Ti/Au PAD metal pattern was formed by performing a lift-off process using an acetone solution. This evaporation and lift-off process methodology resulted in an adhesion problem between the PAD metals and the substrates, as shown in Figure 4a. To solve the problem, the process methodology of the sputtering and metal etching was finally applied, and the detailed process flow was as follows: The cleaning process was conducted as aforementioned. The Ti/Au PAD metal with a thickness of 400/8000 Å was then sputtered using sputter equipment with a predeposition of 10 s, bias power of 700 W, and operating pressure of 10^−6^ Torr. A photolithography process with the same CD of 60 μm was then performed using a positive AZ601 PR and EVG640 contact aligner equipment. The Ti/Au metal was then selectively wet-etched by immersing it in Ti and Au etchants for 50 and 30 s, respectively. The positive PR was removed with acetone-IPA-DI solutions. The PAD metal formed with the sputtering and metal etch process did not have any adhesion problems with the underlying substrate, as shown in Figure 4b.

**Figure 5:**
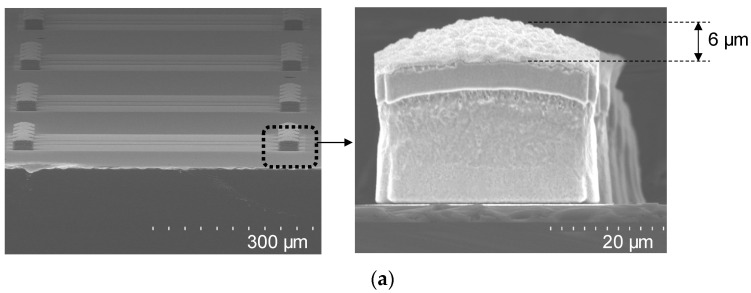

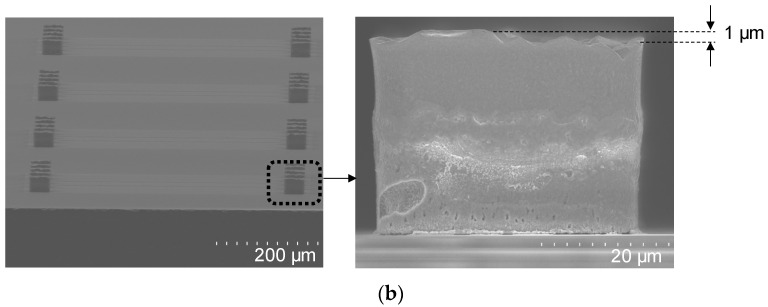
SEM images after formation of microbump (μ-bump) metal: (**a**) μ-bump metal that additives were used in in the Cu electroplating (EP) process; (**b**) μ-bump metal that additives were not used in in the Cu EP process.

The process for the formation of the μ-bump metal was performed on a SiC-substrate-based sample, as seen in the conceptual diagram in Figure 2c. First, a 300/2000 Å thick Ti/Au seed metal was deposited using the aforementioned sputtering equipment, and then a photolithography process for electroplating (EP) was performed using a negative JSR-126N PR, a contact aligner, and a hard-bake process (110 °C for 8 min), leading to a PR thickness of 30 μm or more. An EP process was then conducted, wherein Cu, Ni, and SnAg metals were sequentially deposited to form μ-bumps with a height of 20 μm or more. The thickness of Ni and SnAg metals was set to be more than 2 μm and 6 μm, respectively. The PR and seed metal were then removed by immersing in an STR2000 solution for 30 min at 40 °C and in Ti/Au etchants for a total of 80 s, respectively. It was paramount for the bump to have a flat-top shape to achieve a high-yield performance of the flip-chip bonding. In the EP process of the Cu metal, a copper sulfate solution and additives are generally used. According to our experimental results, when the additives were mixed with the copper sulfate solution, the μ-bump had a convex top shape with a height difference from the top center to the top edge of approximately 6 μm, as shown in Figure 5a, leading to bonding failure. Accordingly, the additives were not used in the Cu EP process to obtain a flat-top shape, as shown in Figure 5b. The copper sulfate solution was provided by ATOTECH.

**Figure 6:**
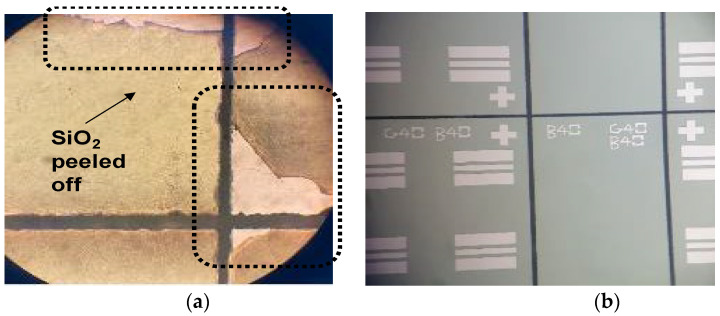
Microscope images after dicing process: (**a**) dicing process where a dielectric removal process was not included; (**b**) dicing process where a dielectric removal process was included.

A dicing process was conducted for the prefabricated InP-substrate-based and SiC-substrate-based samples, as seen in a conceptual diagram of Figure 2d. First, the two samples were protected with a AZ601 PR coating and soft-baking at 100° at 60 s to prevent wafer contamination by particles generated during the dicing process. Second, using the DISCO DFD640 dicing equipment and a KH5-1840 blade, an InP-substrate-based sample with a full size of 3 inches and a SiC-substrate-based sample with a full size of 4 inches were diced to a size of 1 × 1 cm^2^ and 1.1 × 1.1 cm^2^, respectively. Third, the PR was removed and the samples were cleaned using Acetone-IPA-DI solutions. As a result of the process, a peeling problem was found, where the SiO_2_ dielectric layer was peeled off around the dicing lines on the InP substrate, as shown in Figure 6a. The peeling problem was solved by adding a dielectric removal process before the aforementioned dicing process, which selectively removed the dielectric layer around the dicing lines. The dielectric removal process was carried out in the order of a photolithography process using an S700 positive PR and an EVG contact aligner, a hard-baking process for 15 min at 150°, a wet-etching process for 90 min in BOE solution, and a PR removal process using Acetone-IPA-DI. Figure 6b shows an SEM image of the dicing process with the inclusion of the dielectric removal process.

**Figure 7:**
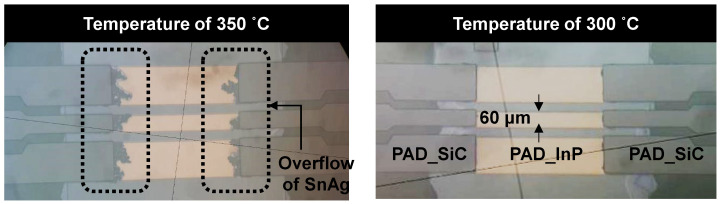
Microscope images of a flip-chip-bonded InP-to-SiC sample with different bonding temperatures of 350 and 300°.

A flip-chip SnAg-to-Au solder bonding process, as seen in the conceptual diagram of Figure 2e, was established by assembling the diced InP-substrate-based and SiC-substrate-based samples. The bonding process was conducted using the DFC-2000C flip-chip bonder equipment with conditions of a bonding pressure of 10 N and a total bonding time of 16.1 s. The bonding temperature was set to 300°, as the overflow phenomenon of SnAg material occurred above 350°, as shown in Figure 7. The alignment error of the flip-chip bonding was within 2 μm.

## 3. Results and Discussion

### 3.1. Performance of Flip-Chip-Bonded InP-to-SiC CPW Lines Consisting of 10 Daisy Chains Interconnected by a Hundred μ-Bumps

**Figure 8:**
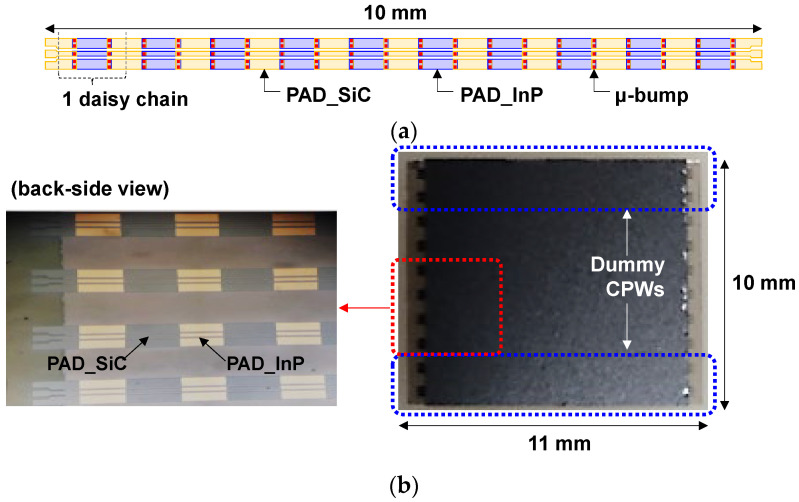
Implementation results of InP-to-SiC CPW lines with a hundred μ-bumps: (**a**) schematic diagram of a InP-to-SiC CPW line; (**b**) microscope images of a fabricated flip-chip-bonded sample with an area of 11 × 10 mm^2^ arranged with ten CPW lines (six real and four dummy lines).

**Figure 9:**
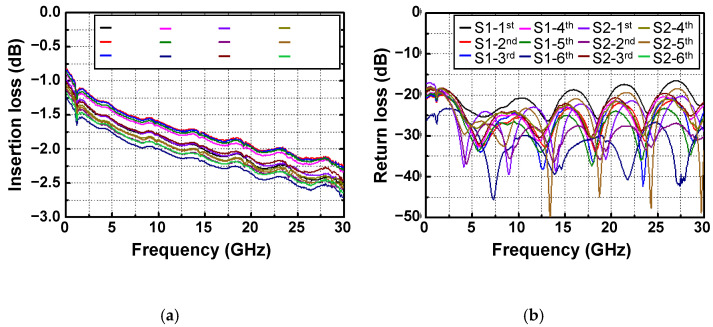
Measured S-parameter results of twelve InP-to-SiC CPW lines with a hundred μ-bumps arranged in two flip-chip-bonded samples: (**a**) insertion loss; (**b**) return loss.

By utilizing the flip-chip μ-bump bonding technology, InP-to-SiC CPW lines where both PAD metals on the InP and SiC substrates were interconnected through μ-bumps were implemented. To pursue the scale-up of the bonding technology for mmW application, an InP-to-SiC CPW line consisted of ten daisy chains interconnected by a hundred μ-bumps, and its length was as high as 10 mm, as shown in Figure 8a. Ten InP-to-SiC CPW lines were arranged in a flip-chip-bonded sample with an area of 11 × 10 mm^2^, as shown in Figure 8b. Among ten CPW lines, the upper two lines and the lower two lines served as dummy patterns for achieving high-yield performance. Two identically designed flip-chip-bonded samples were fabricated. S-parameter data for all real CPW lines arranged in the two samples were measured using the N5225B PNA network analyzer (NA). Figure 9a,b show the measured results for the insertion and return losses of the CPW lines, respectively. The return loss was more than 15 dB over the frequency of 30 GHz from DC. The insertion loss was in the range of 2.24 to 2.71 dB at 30 GHz, and its average value was 0.25 dB/mm, which was comparable to the insertion loss values of previously reported conventional mmW CPW lines without any bonding technologies [19,20,21]. The deviation of the insertion loss for the twelve CPW lines was within ±10%, which verified that the flip-chip-bonded μ-bump process between the InP-to-SiC substrates was well established, exhibiting good uniformity.

**Figure 10:**
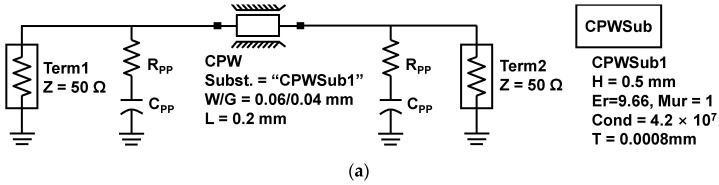

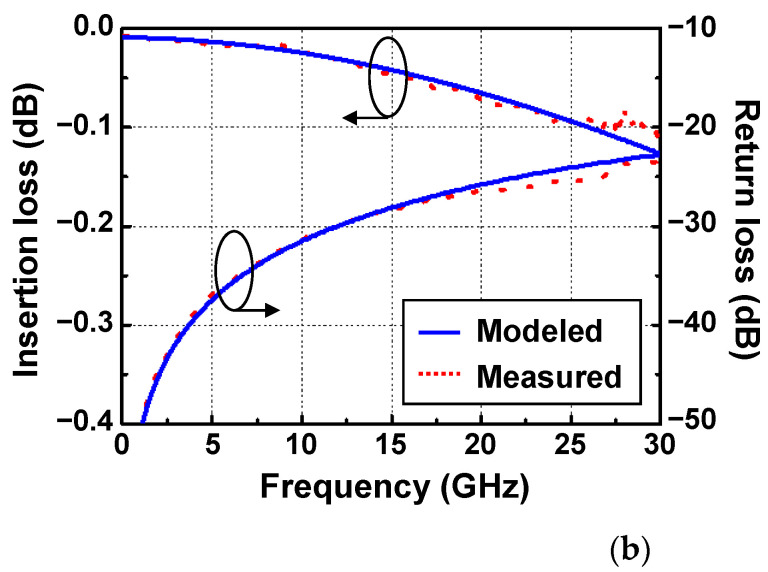
De-embedding for an RF pad region of CPW lines: (**a**) RF modelled circuit diagram of a thru pattern; (**b**) measured and modeled results for S-parameter of the thru pattern.

**Figure 11:**
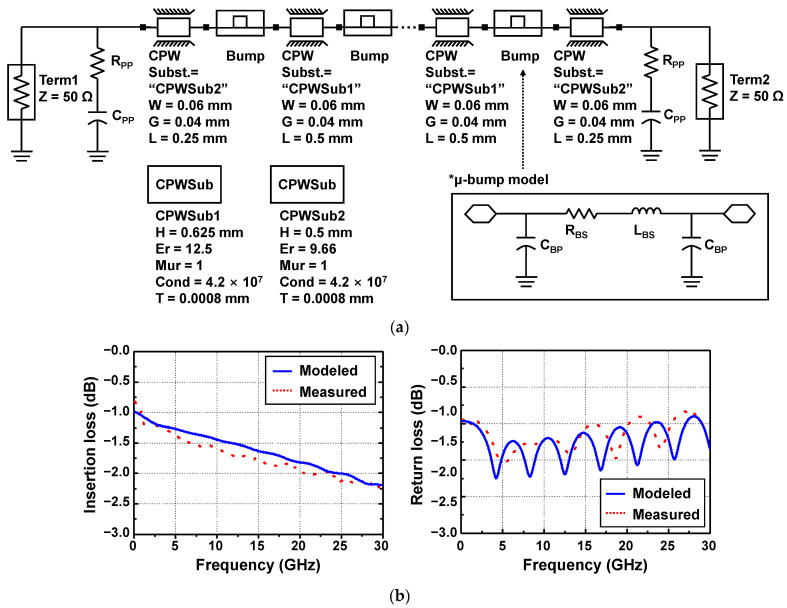
RF modeling of flip-chip-bonded InP-to-SiC CPW lines: (**a**) equivalent circuit diagram for RF modeling of CPW lines; (**b**) measured and modeled S-parameter results for a CPW line with 10 daisy chains.

The RF modeling of fabricated μ-bumps was essential to utilize the flip-chip bonding technology for the mmW application. First, the RF pad region inserted for the RF measurement of CPW lines, as described in Figure 1a, was de-embedded using fabricated open and thru patterns. Figure 10a shows a modeled circuit diagram of a thru pattern with a length of 200 μm, which was drawn in the advanced design system (ADS) simulator. Shunt resistor (R_PP_) and capacitor (C_PP_) devices were used for the proper RF modeling of the pad region. Figure 10b shows measured and modeled results for the S-parameter of the thru pattern. When R_PP_ and C_PP_ were 40 Ω and 12 fF, respectively, the modeled results were in good agreement with the measured results. Next, the RF modeling of the fabricated μ-bump was carried out using measured S-parameter results of the flip-chip-bonded InP-to-SiC CPW line with 10 daisy chains. Figure 11a shows a circuit diagram used for the fabricated CPW lines. As an equivalent circuit model of the fabricated μ-bump, a pi model was used, which consisted of a series resistance (R_BS_), a series inductance (L_BS_), and two shunt capacitors (C_BP_) [22]. Figure 11b shows measured and modeled results of the S-parameter of the fabricated CPW lines with 10 daisy chains. When R_BS_, L_BS_, and C_BP_ were 0.35 Ω, 50 pH, and 20 fF, respectively, the modeled results were well matched with the measured results.

### 3.2. Application to mmW Device of the Flip-Chip μ-Bump Bonding Technology

**Figure 12:**
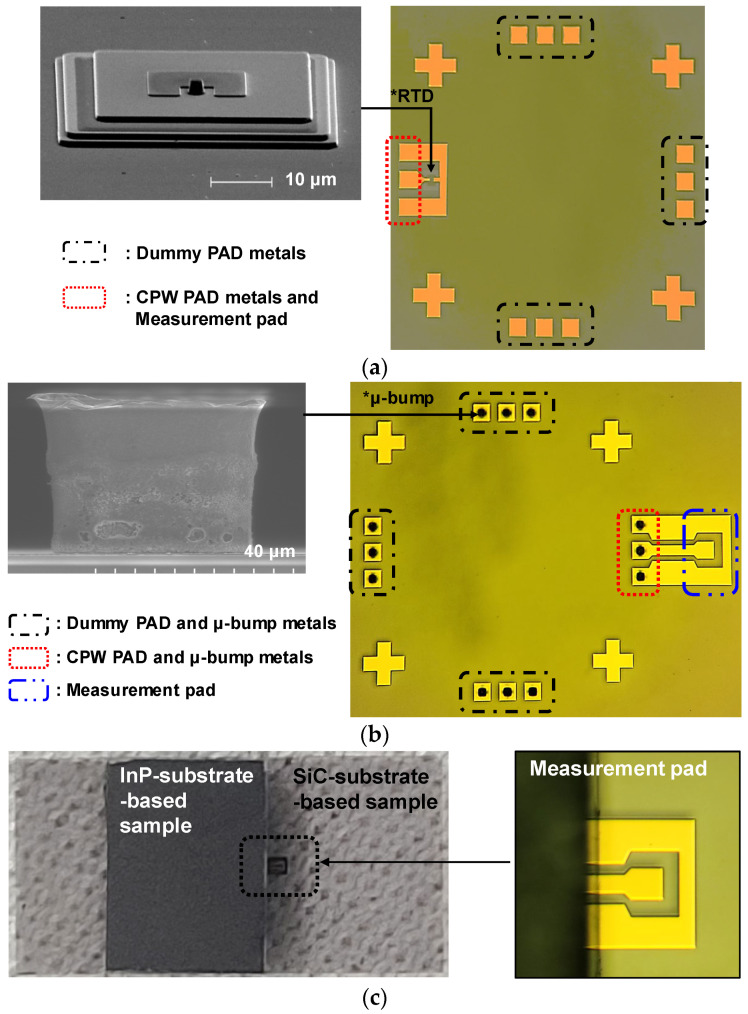
Implementation results of a flip-chip bonded InP-to-SiC resonant tunneling diode (RTD): (**a**) an InP-substrate-based sample; (**b**) a SiC-substrate-based sample; (**c**) a flip-chip-bonded sample with an InP-to-SiC RTD.

To demonstrate the mmW application capability of the flip-chip bonding technology, an InP resonant tunneling diode (RTD), which is one of the semiconductor devices operating at mmW and terahertz (THz) frequencies [23,24], was flip-chip-bonded for the first time with the SiC substrate and its DC, and RF performance was investigated. Figure 12a shows a fabricated InP-substrate-based sample consisting of an RTD, CPW PAD metals for the flip-chip interconnection, and dummy PAD metals functioning as supporting pillars during the bonding process. The inset shows an SEM image before the device passivation process of the fabrication RTD. The epitaxial structure and process sequence of the RTD were described elsewhere [25]. Figure 12b shows a fabricated SiC-substrate-based sample consisting of CPW PAD and μ-bump metals for the flip-chip interconnection, and dummy PAD and μ-bump metals functioning as supporting pillars. Figure 12c shows a microscope image after the InP-substrate-based sample was flip-chip-bonded with the SiC-substrate-based sample.

**Figure 13:**
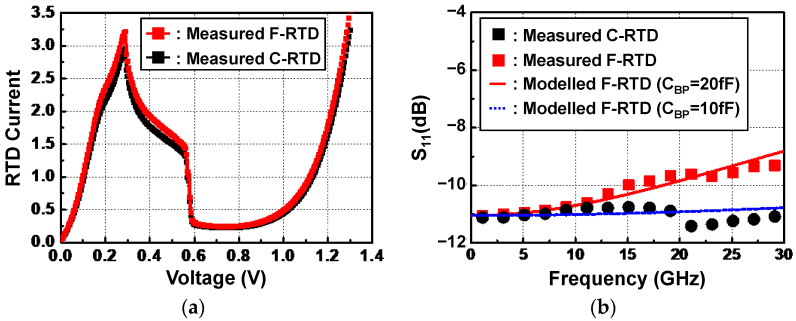
Measurement results of conventional InP resonant tunneling diode (C-RTD) and flip-chip-bonded InP-to-SiC resonant tunneling diodes (F-RTD): (**a**) DC I–V curve; (**b**) S-parameter data. C_BP_ denotes a parasitic shunt capacitance of μ-bump.

A conventional InP RTD without a flip-chip bonding interconnection (C-RTD) and an InP-to-SiC RTD with a flip-chip bonding interconnection (F-RTD) were measured by being probed at measurement pads (seen in Figure 12a,b,) respectively, with respect to the DC and RF characteristics. Figure 13a shows a DC I-V curve of the two RTD devices, which was measured with the Keithley 4200-SCS/F semiconductor characterization system and Summit 11862B probe station. The two RTDs exhibited nearly the same peak and valley voltages of 0.3 and 0.75 V, respectively. The peak and valley currents of F-RTD were 3.21 and 0.24 mA, which were approximately 9 % higher than those (2.94 and 0.22 mA) of C-RTD. This current difference was attributed to the wet-chemical etching variation in the mesa process of the two RTDs [25]. Figure 13b shows measured S-parameter data of the two RTDs measured with the N5225B PNA network analyzer (NA) equipment. The RTDs were biased at 0.2 V. It was observed that the S_11_ value of F-RTD increased compared with that of C-RTD as the frequency increased. This S_11_ increase was mainly attributed to the high C_BP_ value of 20 fF. From the ADS simulation results, based on the aforementioned equivalent model of the bump, the S_11_ graph of F-RTD was the same as that of C-RTD when the C_BP_ value decreased to less than 10 fF from 20 fF, as shown in Figure 13b. C_BP_ was generated from the bump-pad region, corresponding to the region of bump-pad length of ‘a’ in Figure 1. In this work, the bump-pad length for F-RTD was as large as 80 μm, while the bump size for F-RTD (‘b’ in Figure 1) was 40 μm. Because the overflowed SnAg after the flip-chip bonding was present within 5 μm from the edge of the μ-bump metals and the alignment error of bonding equipment was within 2 μm, the bump-pad length for F-RTD could be reduced to less than 60 μm, corresponding to a C_BP_ of 10 fF.

## 4. Conclusions

A process methodology for flip-chip μ-bump bonding between InP and SiC substrates for a mmW wireless communication application was proposed, consisting of a SiO_2_-based dielectric passivation process, a sputtering-based pad metallization process, an EP bump process enabling a flat-top μ-bump shape, a dicing process without the peeling of the dielectric layer, and a SnAg-to-Au solder bonding process. By using the flip-chip bonding process, 10 mm long InP-to-SiC CPW lines with 10 daisy chains interconnected with a hundred μ-bumps were fabricated. All InP-to-SiC CPW lines placed on two samples, one of which had an area of approximately 11 × 10 mm^2^, exhibited uniform performance with insertion loss deviation within ±10% along with an average insertion loss of 0.25 dB/mm, while achieving return losses of more than 15 dB at a frequency of 30 GHz, which were comparable to the insertion loss values of conventional CPW lines. In addition, an InP-to-SiC resonant tunneling diode device was fabricated for the first time and its DC and RF characteristics were investigated.
